# The application of hyaluronic acid-containing artificial tears on the ocular surface of children receiving orthokeratology contact lens treatment

**DOI:** 10.7150/ijms.113380

**Published:** 2025-07-28

**Authors:** Chia-Yi Lee, Shun-Fa Yang, Jing-Yang Huang, Chao-Kai Chang

**Affiliations:** 1 Institute of Medicine, Chung Shan Medical University, Taichung, Taiwan.; 2 Nobel Eye Institute, Taipei, Taiwan.; 3 Department of Ophthalmology, Jen-Ai Hospital Dali Branch, Taichung, Taiwan.; 4 Department of Medical Research, Chung Shan Medical University Hospital, Taichung, Taiwan.; 5 Department of Optometry, Yuanpei University of Medical Technology, Hsinchu, Taiwan.

**Keywords:** hyaluronic acid, orthokeratology contact lens, dry eye, myopia, ocular surface

## Abstract

**Purpose:** The purpose of this study was to compare the effects of the application of hyaluronic acid (HA)-containing artificial tears and non-HA artificial tears on the ocular surface of children receiving orthokeratology contact lens treatment.

**Method:** Charts of patients fitted with orthokeratology contact lenses in any of 20 local clinics were reviewed in this retrospective cohort study. The patients were then categorized according to the artificial tear type used, resulting in 85 and 95 patients being placed into the non-HA and HA groups, respectively. The primary outcomes include fluorescein ocular surface staining and dry eye disease (DED)-related symptoms which were measured at 1, 3 and 6 months after orthokeratology contact lens treatment. An independent t test and Chi-square test were used for statistical analysis.

**Results:** At one month, there was a statistically significant greater number of non-HA patients with staining (14 non-HA, 4 HA, P=0.017). In the final visit, the incidence of ocular surface stain was also significantly lower in the HA group (12 non-HA, 2 HA, P = 0.010). At one month, there was a statistically significant greater number of non-HA patients with DED-related symptoms (24 non-HA, 12 HA, P = 0.029). Finally, the number of DED-related symptoms was significantly lower in the HA group (P = 0.005). After a 6-month follow-up, the spherical equivalent refraction (SER) and axial length (AXL) values between the two groups showed no significant difference (all P > 0.05).

**Conclusions:** The application of HA-containing artificial tears resulted in less ocular surface staining and fewer DED-related symptoms in children wearing overnight orthokeratology contact lenses.

## Introduction

Myopia is a prevalent disease worldwide, and the development of high myopia may cause vision-threatening complications 1. In the Asian population, the overall prevalence of myopia in children may be high, as a prevalence of 95 percent was found in the young population of eastern Asia 2. To control myopia and prevent the development of high myopia, orthokeratology contact lenses have been widely utilized 3. As shown in previous publications, the application of orthokeratology contact lenses can effectively reduce spherical equivalent refraction (SER) progression and retard the rate of axial length (AXL) elongation 4, 5.

Despite the fair ability of orthokeratology contact lenses to control myopia, several complications could still occur after their application 6, 7. Infectious keratitis may be the most dreaded complication of orthokeratology contact lenses, in which severe visual impairment could develop even with proper management 8. In addition, corneal neovascularization may occur after the usage of orthokeratology contact lenses, although the incidence is low 9. Corneal abrasion is another common complication of orthokeratology contact lenses, which can cause irritation and tearing 10, and the lenses may need to be changed to resolve the symptom. Finally, dry eye disease (DED), which is defined as homeostasis loss of the tear film with ocular symptoms 11, can also develop after the utilization of orthokeratology contact lens and leading to further unpleasant symptoms 12.

Artificial tears have been prescribed for children receiving orthokeratology contact lens management to lubricate their ocular surface and reduce cornea-related complications 13. In an earlier study, hyaluronic acid (HA)-containing artificial tears were applied to the eyes of patients who received cataract and refractive surgeries to reduce ocular surface damage 14, 15. Nevertheless, studies have rarely investigated the effectiveness of hyaluronic acid-containing and other artificial tears in terms of reducing the complications of orthokeratology contact lens usage. Because hyaluronic acid-containing artificial tears can reduce inflammation, which is one of the mechanisms of ocular surface injury 16, the effectiveness of hyaluronic acid-containing artificial tears may be different compared to others which need evaluation.

Consequently, the purpose of the current study is to investigate the signs of ocular surface (corneal) damage and DED-related symptoms between hyaluronic acid-containing artificial tears and other artificial tears for orthokeratology contact lens management. The post-treatment outcomes of orthokeratology contact lens management were also evaluated.

## Materials and Methods

### Ethics declaration

All the interventions in this study were conducted in accordance with the 1964 Declaration of Helsinki and its sequential amendments. Additionally, the current study was approved by the Institutional Review Board of the National Changhua University of Education (project code: NCUEREC-113-056; date of approval: 3 July 2024). The need for written informed consent was deferred by the National Changhua University of Education because of the retrospective design of this study.

### Participant selection

This retrospective chart review cohort study was conducted (from 11/02/2023 to 05/02/2024) at the Nobel Eye Institution, which has more than 20 ophthalmic clinics in the Taiwan region. The patients that fulfilled the following criteria were included in the study population: (1) aged 8 to 15 years old; (2) received orthokeratology contact lens treatment at any branch of the Nobel Eye Institute; (3) had a baseline SER from -1.00D to -5.00 diopters (D); and (4) followed up at the Nobel Eye Institute for at least 6 months. In addition, the following exclusion criteria were applied: (1) the receipt of unilateral orthokeratology contact lens management; (2) the application of atropine or other myopic control tools, like dual-focus contact lenses or defocus incorporated multiple segments spectacle lenses; (3) the use of more than one type of artificial tears during the follow-up period; and (4) withdrawal from orthokeratology contact lens management within 6 months from the start due to any reason. All the children were told to wear the orthokeratology contact lenses while sleeping for at least 8 hours, and there was no “holiday” for these orthokeratology contact lens users. The children were then categorized into different groups according to the artificial tears they used. The decision to use artificial tears depended on the parents' choice after a thorough discussion with ophthalmologists. In addition, only the right eye of each child was included in the analysis. Finally, 85 and 95 patients were placed into the non-HA and HA groups, respectively.

### Artificial tears

Two different artificial tears were utilized for the children wearing orthokeratology contact lenses in the current study. In the non-HA group, artificial tears (Selear, Oasis Chemical Industries CO., LTD., Suao, Yilan, Taiwan) containing sodium chloride, potassium chloride, sodium carbonate anhydrous and sodium dihydrogenphosphate were applied. In the HA group, preservative-free HA-containing artificial tears (Systane ® Hydration UD, Alcon, Fort Worth, Texas, United States) consisting of sodium hyaluronate, polyethylene glycol, propylene glycol and hydroxypropyl guar were used. The children and parents were instructed to use the artificial tears twice per day: before putting in the orthokeratology contact lenses and after removing them. Additionally, artificial tear application was recommended if dryness developed at any time, while the non-HA group was told to apply the artificial tears a maximum of five times per day. The limit in the non-HA group is due to the preservatives in the non-HA artificial tear. The children could change the type of artificial tears used during the follow-up period, but they would then be excluded from the current study.

### Ophthalmic examination

The initial data of each child were obtained from medical documents, including information on age, sex, corrected distance visual acuity (CDVA), sphere power, cylinder power, corneal astigmatism extent and AXL. Cycloplegic refraction and AXL tests were conducted using an autorefractor (KR-8900, Topcon, Itabashi-ku, Tokyo, Japan) and a biometry device (IOL Master 500, Carl Zeiss, Göschwitzer Str., Jena, Germany), respectively. The children's baseline simulated keratometry (estimated corneal power) and corneal astigmatism values were obtained using a topographic device (TMS-5, Tomey Corporation, Nishi-Ku, Nagoya, Japan). All these ophthalmic examinations were performed three times, and the average values were used. The SER was calculated as the sphere power plus half of the cylinder power in the current study. Regarding the cycloplegic SER intervention, topical tropicamide (Better eye drop, Aseptic Innovative Medicine Co. Ltd., Taoyuan dist., Taoyuan, Taiwan) was used, and optometrists then evaluated the pupil diameter, and cycloplegic SER was implemented if the pupil diameter was greater than 8 mm. The eyes of all the patients in the current study were measured with the same device, and the same physician followed up with them during the follow-up period. Regarding the condition of the ocular surface, ocular staining was performed by applying a fluorescein strip onto the lower bulbar conjunctival region; the children were then told to close their eyes for about 5 seconds, and the staining status was evaluated by a physician under a slit-lamp biomicroscope. The grade of ocular surface staining was in accordance with the Oxford Scheme (range from 0 to 5) outlined in a previous publication 17. For DED-related symptoms, the symptoms in the Ocular Surface Disease Index questionnaire (range from 0 to 100) were evaluated by the ophthalmologists at every visit. Of note, we only used the symptom items rather than the scoring system of the OSDI, thus we ended up counting the number of symptoms in the current study (0-5). The status of SER, AXL, ocular surface staining and DED-related symptoms was evaluated before and 1, 3 and 6 months after orthokeratology contact lens usage. The children's orthokeratology contact lens usage compliance was evaluated by the ophthalmologists, information on which was obtained from the children's parents at every visit, and all the children obeyed the therapeutic program in accordance with their parents' reminders.

### Statistical analysis

SPSS version 20.0 (SPSS Inc., Chicago, Illinois, USA) was employed for all the statistical analyses described in the current study. The statistical power of the current study was 0.96, with a 0.05 alpha value and a medium effect size, which was created via G∗power version 3.1.9.2 (Heinrich Heine Universität at Düsseldorf, Germany). The Shapiro-Wilk test was employed to examine the normality of the data in study populations, and the results revealed that there were normal distributions for all initial data (all P > 0.05). Descriptive analysis was employed to present the baseline data of the non-HA and HA groups. An independent t test and Chi-square test were employed to analyze the differences in the baseline data between the two groups. Another independent t test was then applied to compare the post-treatment CDVA, SER and AXL values between the two groups. We divided the ocular surface stain condition into absent (grade 0), mild (grade 1-2) and prominent (grade 3 or more), and the severity of ocular surface staining between the two groups was analyzed using a Chi-square test. In the next step, we divided the number of DED-related symptoms into no symptoms, 1-2 symptoms and 3 or more symptoms, and the number of DED-related symptoms in the two groups was analyzed using a Chi-square test. Statistical significance was indicated by a P value < 0.05 in the current study.

## Results

The baseline characteristics of the two groups are shown in Table 1. The mean age was 10.78±1.52 and 10.53±1.61 in the non-HA and HA groups, respectively, without a significant difference (P = 0.287). In addition, the sex distributions between the two groups also showed an insignificant difference (P = 0.876). All ophthalmic parameters, including CDVA, cycloplegic refraction, AXL, simulated keratometry and corneal astigmatism, demonstrated an insignificant difference between the non-HA and HA groups (all P > 0.05). For the ocular surface conditions, there was no ocular surface staining or DED-related symptoms in either the non-HA or HA group before orthokeratology contact lens treatment (both P > 0.05) (Table 1).

There was no ocular surface staining found before the orthokeratology contact lens treatment in the two groups. At one month, there was a statistically significant greater number of non-HA patients with staining (14 non-HA, 4 HA, P=0.017). The non-HA groups continued to have higher numbers of patients who presented with staining at 6 months (12 non-HA, 2 HA, P = 0.010) (Table 2). Similarly, no children reported DED-related symptoms before the orthokeratology contact lens treatment in either group. At one month, there was a statistically significant greater number of non-HA patients with DED-related symptoms (24 non-HA, 12 HA, P = 0.029). At the last follow-up, the number of patients who reported any symptoms was still significantly lower in the HA group (0 symptoms: 70 and 92 in non-HA and HA; 1-2 symptoms: 14 and 3 in non-HA and HA; 3 or more symptoms: 1 and 0 in non-HA and HA; P = 0.005) (Table 3).

The initial SER values were -2.78±0.99 and -2.81±0.87 D in the non-HA and HA groups, respectively. After the 6-month follow-up period, the SER values increased to -2.92±0.95 and -2.93±0.87 D in the non-HA and HA groups, respectively. The SER values between the two groups showed no significant difference throughout the follow-up period (all P > 0.05) (Table 4). The baseline AXL was 24.28±1.36 and 24.31±1.31 mm in the non-HA and HA groups, respectively. After the 6-month follow-up period, the AXL increased to 24.33±1.40 and 24.35±1.32 mm in the non-HA and HA groups, respectively. The AXL values between the two groups had no significant difference at all points (all P > 0.05) (Table 4).

## Discussion

In short, the current study showed that there were fewer patients presenting with corneal staining in the HA group and fewer patients presenting with DED-related symptoms in the HA group after orthokeratology contact lens treatment. Additionally, the orthokeratology contact lens treatment demonstrated similar SER and AXL control effects regardless of the type of artificial tears used.

HA-containing artificial tears have been applied in the field of ophthalmology with several benefits 18, 19. The high-molecular-weight HA added into artificial tears is an anti-inflammatory substance that can reduce the inflammatory response 20. In a previous study, the concentration of matrix metalloproteinases decreased after the application of HA 21. In another study, the application of HA effectively suppressed the levels of inflammatory biomarkers on the ocular surface, including interleukin and tumor necrosis factor 22. In addition to HA's anti-inflammatory effects, the usage of HA can significantly lower oxidative stress on the ocular surface 23. Moreover, the high viscosity of HA can reduce the friction of the ocular surface compared to the saline solution used in an experimental study 24, and the utilization of HA can accelerate the corneal wound healing process 25. Regarding clinical research, the application of HA-containing artificial tears was shown to significantly retard the severity and progression of DED in a review article 26. As for ophthalmic surgery, the application of HA-containing artificial tears is associated with fewer postoperative cataract surgery symptoms than non-HA artificial tears 14. Additionally, the application of HA-containing artificial tears would contribute to the faster healing of the corneal epithelium in patients receiving refractive surgery 27. Orthokeratology contact lenses, although having high oxygen permeability 7, can cause ocular surface damage due to an increase in inflammation 28. Moreover, post-treatment DED after orthokeratology contact lens usage has been reported 12. Because HA-containing artificial tears can benefit the ocular surface and the application of orthokeratology contact lenses may exert negative effects on the ocular surface, we speculate that the usage of HA-containing artificial tears may reduce the complications of orthokeratology contact lens treatment. This concept was at least partially supported by the results of the current study.

The application of HA-containing artificial tears is correlated with a lower incidence of ocular surface staining after orthokeratology contact lens treatment. In the preceding literature, artificial tears were applied for orthokeratology contact lens treatment to reduce corneal damage 29. In addition, the utilization of antibiotic agents could reduce the risk of infectious keratitis during orthokeratology contact lens usage 30. Nevertheless, there is no research that evaluates the influence of different types of artificial tears on the postoperative complications of orthokeratology contact lenses. To our knowledge, the current study may be one of the first to represent the correlation between the utilization of HA-containing artificial tears and the lower incidence of corneal fluorescein staining after orthokeratology contact lens usage. In addition, there was no ocular surface damage noted in the study population before the orthokeratology contact lens treatment; thus, the baseline corneal condition may be analogous among all the children in the current study. Furthermore, the average SER and AXL values before orthokeratology contact lens treatment revealed an insignificant difference between the non-HA and HA groups, which may indicate that the planned compression effects of orthokeratology contact lenses on the ocular surface may be similar in the two groups. Consequently, the application of HA-containing artificial tears may be an independent protective factor for the ocular surface complications of orthokeratology contact lens treatment. Importantly, we grade the corneal surface with fluorescein. Moreover, the eyes of three patients developed prominent ocular surface staining in the non-HA group, while no prominent ocular surface staining was found in the HA group. This finding may indicate that the usage of HA-containing artificial tears could reduce the risk of advanced corneal injury in orthokeratology contact lens users. In addition to the HA component, the hydroxypropyl guar included in the HA-containing artificial tears used in the current study could decrease the desiccation levels of corneal epithelial cells and tissue surface friction 31. The combination of HA and hydroxypropyl guar also resulted in higher corneal epithelial cell survival than HA or hydroxypropyl guar alone 32. Thus, hydroxypropyl guar may also contribute to the lower rate of positive ocular surface staining in the HA group compared to the non-HA group.

Regarding symptoms, the frequency of DED-related symptoms was significantly lower in the children receiving orthokeratology contact lens treatment and applying HA-containing artificial tears. In a previous study, the development of DED-related symptoms, including foreign body sensations, irritation, ocular pain and dryness, was not uncommon after orthokeratology contact lens treatment 33. However, there are scant studies that report on methods for reducing the number of symptoms after orthokeratology contact lens usage. In the current study, the rate of DED-related symptoms was about 28 and 12 percent in the non-HA and HA groups, indicating a significant difference between the two groups. Since the viscosity and lubrication of HA-containing artificial tears are better than those of other types of artificial tears 32, a lower friction rate and degree of friction may be associated with a lower rate of DED-related symptoms, as presented in the current study. Furthermore, the numbers children reported 3 or more DED-related symptoms were 3-folds higher in the non-HA group than the HA group one month post-treatment. Finally there were 1 eye in the non-HA group described 3 DED-related symptoms while no children in the HA group described 3 or more DED-related symptoms. The above results may further support the hypothesis that HA-containing artificial tears can reduce orthokeratology contact lens-related complications. Compliance is a significant issue when using orthokeratology contact lenses 34. Because children generally have low tolerance, they may refuse to wear orthokeratology contact lenses if ocular discomfort develops. As a result, it is crucial to reduce post-treatment ocular discomfort in orthokeratology contact lens treatment, and HA-containing artificial tears may be beneficial in this field. On the other hand, although the possibly lower frequency of artificial tear instillation in the may correlated with DED signs and symptoms, the additional boric acid (preservatives) in the non-HA artificial tear may also cause ocular surface irritation and subsequent DED signs and symptoms 35. Thus, the frequency of non-HA artificial tear instillation may not affect the result prominently. Regarding the compliance of our patients, the compliance was monitored by their parents and all the children actually wore the orthokeratology contact lens according to their parents. Still, the HA-containing artificial tears they used was preservative-free daily-disposable type, thus we cannot collected and measured them to ensure correct usage.

There were certain limitations in the current study. Firstly, the retrospective design of the current study reduced the homogeneity of the non-HA and HA populations, although all the baseline parameters did not demonstrate significant differences between the two groups. Secondly, postoperative topographic examinations were not performed due to the retrospective nature of this study; thus, whether ocular irritation and ocular surface damage resulted from the dislocation of orthokeratology contact lenses could not be confirmed. In addition, all the children that received orthokeratology contact lens treatment at our institution used artificial tears because of safety concerns. Accordingly, a control group that received orthokeratology contact lens treatment without artificial tear usage could not be established to evaluate the influence of artificial tears more carefully. Finally, all the children enrolled in the current study are Han Taiwanese, so the external validity of the current study may be diminished.

In conclusion, the application of HA-containing artificial tears could reduce the incidence of ocular surface damage during orthokeratology contact lens treatment compared to non-HA artificial tears. Furthermore, DED-related symptoms also developed less frequently in the patients using HA-containing artificial tears. Consequently, the application of these tears may be considered for those scheduled for orthokeratology contact lens treatment. Further large-scale prospective studies to evaluate the long-term effects of HA-containing artificial tears on orthokeratology contact lens treatment are mandatory.

## Figures and Tables

**Table 1:** Baseline characteristics between the two groups.

Characteristics	Non-HA group (N = 85)	HA group (N = 95)	P
Age (mean ± SD)	10.78±1.52	10.53±1.61	0.287
Sex (male-female)	54:31	62:33	0.876
CDVA (LogMAR)	0.00±0.00	0.00±0.00	0.999
Cycloplegia refraction			
Sphere	-2.34±1.13	-2.41±1.04	0.666
Cylinder	-0.87±0.46	-0.80±0.49	0.326
SER	-2.78±0.99	-2.81±0.87	0.829
AXL	24.28±1.36	24.31±1.31	0.880
Simulated keratometry	43.25±2.34	43.37±2.47	0.739
Corneal astigmatism	-1.15±0.68	-1.06±0.73	0.395
Ocular surface stain			0.999
Absent	85	95	
Mild	0	0	
Prominent	0	0	
DED-related symptoms			0.999
0	85	95	
1-2	0	0	
≥ 3	0	0	

AXL: axial length; CDVA: corrected distance visual acuity; DED: dry eye disease; HA: hyaluronic acid; SD: standard deviation; SER: spherical equivalent refraction.

**Table 2:** The ocular surface stain status between the two groups.

Parameter	Non-HA group	HA group	P
Pre-treatment			0.999
Absent	85	95	
Mild	0	0	
Prominent	0	0	
1 month post-treatment			0.017*
Absent	71	91	
Mild	11	4	
Prominent	3	0	
3 months post-treatment			0.006*
Absent	72	93	
Mild	12	2	
Prominent	1	0	
6 months post-treatment			0.010*
Absent	73	93	
Mild	11	2	
Prominent	1	0	

HA: hyaluronic acid. * denotes significant difference between groups.

**Table 3:** The presence of dry eye disease-related symptoms between the two groups.

Parameter	Non-HA group	HA group	P
Pre-treatment			0.999
0	85	95	
1-2	0	0	
≥ 3	0	0	
1 month post-treatment			0.029*
0	61	83	
1-2	18	10	
≥ 3	6	2	
3 months post-treatment			0.027*
0	69	89	
1-2	14	6	
≥ 3	2	0	
6 months post-treatment			0.005*
0	70	92	
1-2	14	3	
≥ 3	1	0	

HA: hyaluronic acid. * denotes significant difference between groups.

**Table 4:** The effects of orthokeratology contact lenses on controlling myopia between the two groups.

Parameter	Non-HA group	HA group	P
SER			
Pre-treatment	-2.78±0.99	-2.81±0.87	0.829
1 month post-treatment	-2.78±1.01	-2.82±0.85	0.773
3 months post-treatment	-2.85±0.96	-2.86±0.88	0.942
6 months post-treatment	-2.92±0.95	-2.93±0.87	0.941
AXL			
Pre-treatment	24.28±1.36	24.31±1.31	0.880
1 month post-treatment	24.26±1.40	24.31±1.35	0.808
3 months post-treatment	24.30±1.41	24.32±1.34	0.922
6 months post-treatment	24.33±1.40	24.35±1.32	0.922

AXL: axial length; HA: hyaluronic acid; SER: spherical equivalent refraction.

## Abbreviations

AXL: axial length
CDVA: corrected distance visual acuity
D: diopter
DED: dry eye disease
HA: hyaluronic acid
SER: spherical equivalent refraction
